# A single-center, randomized, non-inferiority study evaluating seroma formation after mastectomy combined with flap fixation with or without suction drainage: protocol for the *S*eroma reduction and dr*A*in f*R*ee m*A*stectomy (SARA) trial

**DOI:** 10.1186/s12885-020-07242-0

**Published:** 2020-08-07

**Authors:** Lisa de Rooij, Sander M. J. van Kuijk, Els R. M. van Haaren, Alfred Janssen, Yvonne L. J. Vissers, Geerard L. Beets, James van Bastelaar

**Affiliations:** 1 Department of Surgery, Zuyderland Medical Center, Postbus 5500, 6130 MB Sittard, the Netherlands; 2 Department of Clinical Epidemiology and Medical Technology Assessment, Maastricht University Medical Center, Maastricht, the Netherlands; 3 Department of Surgery, Netherlands Cancer Institute, Amsterdam, the Netherlands; 4 GROW School for Oncology and Developmental Biology, University of Maastricht, Maastricht, the Netherlands

**Keywords:** Mastectomy, Seroma, Drain free, Flap fixation

## Abstract

**Background:**

Seroma formation is a common complication after breast cancer surgery and can lead to delayed wound healing, infection, patient discomfort and repeated visits to the outpatient clinic. Mastectomy combined with flap fixation is becoming standard practice and is currently combined with closed-suction drainage. There is evidence showing that closed-suction drainage may be insufficient in preventing seroma formation. There is reasonable doubt whether there is still place for closed-suction drainage after mastectomy when flap fixation is performed.

We hypothesize that mastectomy combined with flap fixation and closed suction drainage does not cause a significant lower incidence of seroma aspirations, when compared to mastectomy and flap fixation alone. Furthermore, we expect that patients without drainage will experience significantly less discomfort and comparable rates of surgical site infections.

**Methods:**

This is a randomized controlled trial in female breast cancer patients undergoing mastectomy and flap fixation using sutures with or without sentinel lymph node biopsy (SLNB). Patients will be eligible for inclusion if they are older than 18 years, have an indication for mastectomy with or without sentinel procedure. Exclusion criteria are modified radical mastectomy, direct breast reconstruction, previous history of radiation therapy of the unilateral breast, breast conserving therapy and inability to give informed consent. A total of 250 patients will be randomly allocated to one of two groups: mastectomy combined with flap fixation and closed-suction drainage or mastectomy combined with flap fixation without drainage. Follow-up will be conducted up to six months postoperatively. The primary outcome is the proportion of patients undergoing one or more seroma aspirations. Secondary outcome measures consist of the number of invasive interventions, surgical site infection, quality of life measured using the SF-12 Health Survey, cosmesis, pain and number of additional outpatient department visits.

**Discussion:**

To our knowledge, no randomized controlled trial has been conducted comparing flap fixation with and without closed-suction drainage with seroma aspiration as the primary outcome. This study could result in finding evidence that supports performing mastectomy without closed-suction drainage.

**Trial registration:**

This trial was approved by the medical ethical committee of Zuyderland Medical Center METC-Z on 20 March 2019 (METCZ20190023). The SARA Trial was registered at ClinicalTrials.gov as per July 2019, Identifier: NCT04035590.

## Background

Seroma formation can be a bothersome complication after breast cancer surgery and is often considered to be an inevitable consequence of mastectomy [1–4]. Seroma can lead to delayed wound healing, infection, skin flap necrosis, patient discomfort and repeated visits to the outpatient clinic. Therefore, extensive research has focused on the pathophysiology to be able to better prevent seroma. Seroma is a collection of serous fluid containing blood plasma and/or lymph fluid and a key element in the prevention seems the obliteration of dead space [4–8]. However, controversy exists about the best technique to achieve this goal. A recent systematic review of flap fixation techniques in reducing seroma formation and its sequelae after mastectomy by van Bastelaar et al. (2018) showed that flap fixation reduces seroma formation and seroma aspiration after mastectomy with or without axillary clearance [9]. Flap fixation with tissue glue appears to yield similar results when compared to flap fixation using sutures [10]. This conclusion is supported by an interim analysis of a trial by Granzier et al. (2019), showing that flap fixation with either sutures or adhesive tissue glue reduces the number of needle aspirations compared to patients undergoing simple wound closure [11]. Other studies on flap fixation also show promising results in reducing the incidence of seroma and seroma aspirations after breast cancer surgery [12–18]. Mastectomy combined with flap fixation is becoming standard practice, and is currently still combined with closed-suction drainage.

For many years closed suction drainage was considered the most effective intervention for reducing seroma formation after breast cancer surgery. This practice was supported by trials showing that mastectomy without the use of drains was associated with a higher occurrence of seroma and greater volumes of seroma in the post-operative period [19–23]. Other studies in drain free mastectomy have shown that application of suction drainage does not prevent seroma formation and that there is no significant difference between the incidence of seroma formation [24–26]. These studies also concluded that closed suction drainage is associated with a longer postoperative hospital stay on average, and that a long duration of drainage was related to a higher incidence of seroma formation [27–29]. The question therefore is if there is still a place for closed-suction drainage after mastectomy with flap fixation, as this has shown to decrease seroma formation [21, 22]. However, the evidence consists of only small case series and a large randomized controlled trial is needed to provide definitive proof.

To our knowledge, no randomized controlled trial has been conducted comparing flap fixation with and without the use of closed-suction drainage using seroma aspirations as the primary outcome. The SPIRIT Statement guidelines were used for designing and describing this trial [30, 31].

## Objectives

### Research hypothesis

We hypothesize that flap fixation with closed suction drainage does not cause a significantly lower incidence of seroma aspirations when compared to flap fixation alone. We also expect that patients without drainage will experience less discomfort and will have comparable rates of surgical site infections.

### Study objectives

#### Primary objective

To evaluate whether omitting drains after mastectomy in combination with flap fixation does not lead to a higher incidence of seroma formation and seroma aspirations in women with invasive breast cancer or ductal carcinoma in situ (DCIS).

#### Secondary objectives

Assess whether patients with or without drainage after mastectomy experience more pain, experience different quality of life, undergo more invasive interventions related to seroma, develop more surgical site infections or require more unplanned visits to the hospital. Self-assessed cosmesis will also be evaluated.

## Methods/design

### Study design

The SARA (Seroma reduction and drAin fRee mAstectomy) trial is a single center randomized controlled trial. As it is obvious whether or not a drain is used, no blinding can be performed. Patients will be allocated to one of two groups. One group will undergo mastectomy combined with flap fixation and placement of low vacuum drainage and the other group will undergo mastectomy with flap fixation without placement of low vacuum drainage. In both groups the same method for flap fixation will be applied. Flap fixation will be achieved using sutures [10].

### Setting

This single center randomized controlled non-inferiority trial will be conducted in a Dutch teaching hospital in the south of the Netherlands, the Zuyderland Medical Center. Roughly 500 patients are treated for breast cancer annually. All patients will be recruited from the surgical breast cancer clinic (Borstcentrum Zuyd) after evaluation for invasive breast cancer or DCIS (ductal carcinoma in situ).

### Eligibility criteria

#### Inclusion criteria

Patients are eligible for inclusion if they meet the following requirements: (1) patients with an indication for mastectomy with or without sentinel lymph node biopsy (SLNB) suffering from invasive breast cancer or DCIS, (2) female gender, (3) older than 18 years.

#### Exclusion criteria

The exclusion criteria are as follows: (1) patients undergoing modified radical mastectomy, (2) patients undergoing breast conserving therapy, (3) patients undergoing direct breast reconstruction, (4) patients with a history of radiation therapy of the unilateral breast, (5) patients unable to comprehend implications and extent of the study and therefore unable to sign for informed consent.

### Recruitment

The trial is planned to start inclusion in January 2020. Patients will be screened in the outpatient department by one of the breast surgeons. If they meet inclusion and exclusion criteria they will be informed about the trial and will be given a week to decide whether or not they want to participate. If so, written informed consent will be obtained.

### Study interventions

**Fig. 1:**
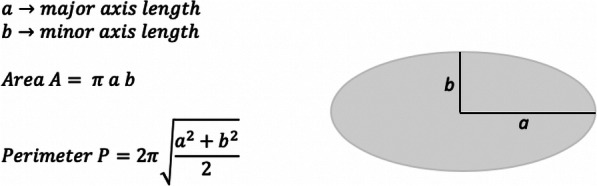
Formula to calculate wound surface

The operative procedure will be performed by one of four experienced breast surgeons. Dissection of the breast tissue including the prepectoral fascia is performed using electrocautery. After performing the mastectomy the extent of the skin flaps will be measured in centimeters from medial to lateral and from cranial to caudal. Wound surface is assumed to be elliptical and will be calculated using the formula A = a x b x π, see Fig. 1. The skin flaps will be sutured on to the pectoral muscle using polyfilament absorbable sutures (Vicryl 3.0), placed at 4–5 cm intervals in two or three rows, depending on the extent of the skin flaps. The distance between all sutures will be 4–5 cm. The fat of the subcutis of the skin flaps will be sutured on to the pectoral muscle. This will be performed as tension free as possible minimizing the risk of dimpling in the skin. The axillary area is not approximated using sutures. The skin edges will be sutured in one layer using absorbable monofilament sutures (Moncryl 3.0 or V-loc 30 cm), depending on the surgeon’s preference. Depending on randomization, a low suction drain will be placed before wound closure (Armstrong medical).

### Mastectomy with low vacuum drainage

In the group with closed suction drainage, one low vacuum drain will be placed in the mastectomy gutter, lateral to the pectoral muscle before flap fixation is performed. The drain is connected to a low suction drain bottle. Drain output will be recorded daily. Drain removal will be performed once daily output is below 50 mL or after a maximum of 48 h, irrespective of drain output.

### Mastectomy without low vacuum drainage

In the group without closed suction drainage the procedure is performed as described in ‘Study interventions’ and placement of drains is omitted.

### Study outcomes

#### Primary outcome measure

The primary outcome is the percentage of patients undergoing seroma aspiration of clinically significant seroma during the first 6 months post-operatively. Clinically significant seroma is defined as:

a. Wound healing is at risk due to seroma (wound dehiscence, seroma leakage, necrosis).

b. There is discomfort or pain caused by large amounts of seroma, characterized by tenseness of the skin.

c. There is contaminated or infected seroma and aspiration is necessary to treat infection. All patients that undergo seroma aspiration due to infection will also be treated with a 1 week course of Augmentin 625 mg 3 times daily.

Seroma presents itself in many forms, ranging from minimal seromas to high volume seromas. It is important to realize that not every seroma is a clinically significant seroma. By defining the primary outcome as stated above, we find this to be the most objective assessment of clinically significant seroma as this is defined by clinical relevance. By clearly stating the definition of the primary endpoint, we hope to avoid misinterpretation and deviation from the study protocol. This creates uniformity in deciding whether seroma needs to be aspirated, which is of crucial importance for assessing the primary endpoint.

#### Secondary outcome measures

Assess:

1. Number of invasive interventions related to seroma or wound healing defined as: every aspiration of clinically significant seroma, incision and drainage of abscess or infected seroma and/or operative debridement of the wound.
2. Surgical site infection (SSI) rate, defined as redness, pain, heat or swelling at the site of the incision or by the drainage of pus. Infection rate will be measured by A) the need for antibiotics, B) seroma aspiration due to infection or C) surgical drainage.
3. Cosmesis assessed by the patient at every planned outpatient clinic visit. At these visits patients are asked to rate the cosmesis of the chest wall using a numeric rating scale (NRS) on a scale from 0 to 10.
4. Quality of life measured using the SF-12 (Short Form) Health Survey. This is a 12-item questionnaire used to assess health related quality of life from the patient’s perspective. Two subscales are derived from the SF-12: the Physical Component Summary (PCS) and the Mental Component Summary (MCS) with a range from 0 to 100. A score of less than 50 means the patient scores lower than the general population and a score of more than 50 means the patient scores higher than the general population. Patients will be asked at baseline and at every postoperative planned outpatient clinic visit to fill in this questionnaire.
5. The number of outpatient department visits, measured during the first 6 months postoperatively. This will be measured to see if patients have to come to the hospital more often due to seroma or seroma related complications.
6. Wound pain and pain at the drain site measured by the patient using the NRS on a scale from 0 to 10 at the first postoperative outpatient clinic visit.

All outcome measures will be measured during the first 6 months postoperatively.

### Follow-up

**Fig. 2:**
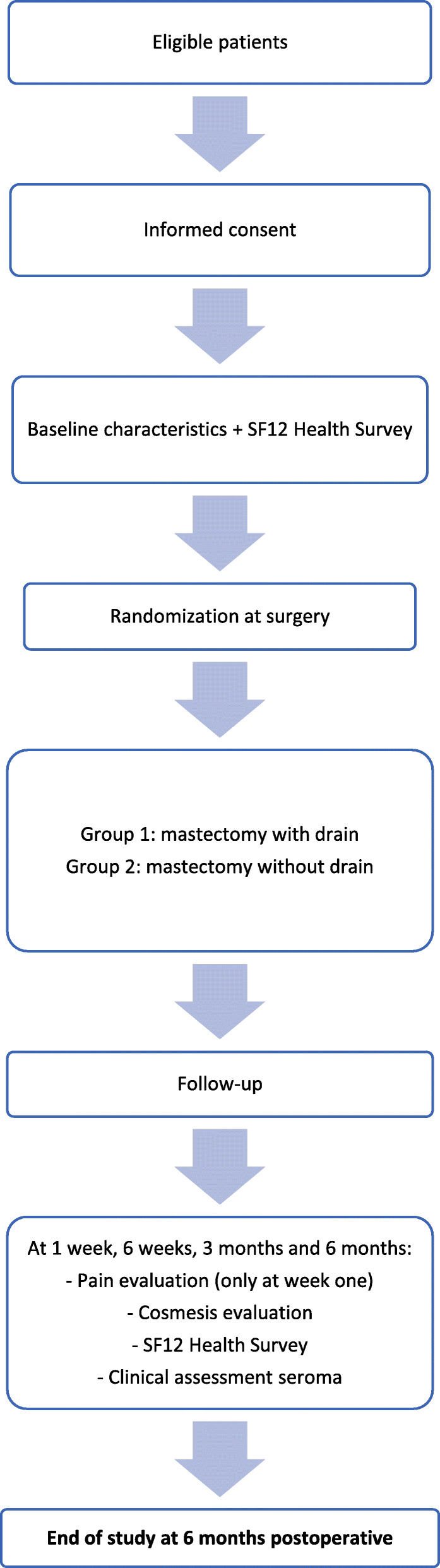
Participant timeline SARA trial

The follow-up period will be 6 months after mastectomy. Patients will visit the outpatient department for evaluation at 1 week, 6 weeks, 3 months and 6 months. See Fig. 2 for the participant timeline. Table 1 represents the time schedule of enrolment, interventions and assessments.

**Table 1:** Time schedule of enrolment, interventions and assessments

	STUDY PERIOD						
	Enrolment	Allocation	Post-allocation				
**TIMEPOINT**	***-t***_***1***_	**0**	***Day 1***	***1 wk.***	***6 wk.***	***3*** ***mos.***	***6 months***
**ENROLMENT:**							
**Eligibility screen**	X						
**Informed consent**	X						
**Allocation**		X					
**INTERVENTIONS:**							
**Mastectomy with drainage**		X					
**Mastectomy without drainage**		X					
**ASSESSMENTS:**							
***Baseline characteristics***	X	X					
***Clinical assessment seroma and complications***				X	X	X	X
***Invasive interventions***			X	X	X	X	X
***SSI***				X	X	X	X
***Cosmesis***				X	X	X	X
***Additional OPD visits***				X	X	X	X
***Wound pain***				X			

### Sample size

Based on previous studies conducted with a follow-up period of 1 year we estimate that about 10% of women in each group require seroma aspirations. If the rate of women requiring aspirations in the drain free group would be 10% larger than the overall aspiration rate of 10%, we would conclude that drain free mastectomy is inferior to standard mastectomy (i.e., the non-inferiority limit is 10%). To be able to have 80% power to reject the null-hypothesis that drain free mastectomy is inferior if in reality it is not, we need to include at least 112 patients per group. To account for a potential drop-out rate of 10% we will include 125 patients per group, or 250 in total. We expect that enrolment of these patients for the study will take 18 months.

### Interim analysis

An interim analysis will be conducted after half of the patients (112) are included. The purpose of this interim analysis is to assess whether the assumptions that have been used to inform the sample size calculation were correct (i.e., the rate of aspirations and the drop-out rate). The total sample size may be adjusted as a result of the interim analysis, thereby ensuring sufficient power to reject the null-hypothesis that drain free mastectomy is inferior at the end of the study. No primary or secondary outcome measures will be tested in the interim analysis, and the results will not contribute to the conclusions of the study.

### Randomization

Patients will be randomly assigned to one of the two groups with a 1:1 allocation as per a computer generated randomization schedule. Randomization will take place on the day of surgery, 30 min prior to wound closure. Patients will be randomized using block randomization with block sizes of 4 and allocated to one of two groups: (1) mastectomy with drain or (2) mastectomy without drain. Due to visibility of the drain no blinding is possible.

### Concealment mechanism

Participants will be randomized using ‘ResearchManager’, which is a data management program used by Zuyderland Medical Center. Allocation concealment will be ensured, as the generated randomization schedule is protected with a password. One surgeon not involved in the treatment of these patients will have access to this password.

### Blinding

As stated above, no blinding is possible due to the visibility of the drain.

### Data collection

The baseline characteristics and demographics will be collected preoperatively after the patient has given written informed consent. In this session the first SF-12 Health Survey questionnaire will be completed by the patient, with or without guidance of a member of the study. At the planned postoperative outpatient clinic visits at 1 week, 6 weeks, 3 months and 6 months, the patients will fill in the SF-12 Health Survey and will be asked to report on the cosmesis and experienced pain. Physical examination will take place to evaluate the wound and the presence of clinically significant seroma.

### Data management

Study data is recorded using a data management program containing case report forms (CRF’s). Data will be updated on a weekly basis. When data is exported, data is anonymized and patients can only be identified by an allocated sequence number. A coordinating investigator will verify data regularly and will check for and correct missing or inconsistent data.

### Statistical analysis

Baseline characteristics of the patients will be described in detail stratified by allocation. Continuous variables will be reported as mean and standard deviation or, in case of severe skewness, as median and interquartile range. Categorical variables will be reported as count and percentage.

Missing data will be imputed to prevent a loss of statistical precision and to reduce the likelihood of biased estimates of treatment effect. To do so, we will select a proper imputation method after careful consideration of the proportion of incomplete patients and the likely reasons for incomplete data.

#### Primary study parameter(s)

The upper bound of the confidence interval of the difference in proportion of patients that received one or more seroma aspirations during the first 6 months post-operatively will be compared to the non-inferiority limit of 10%. In case the upper bound is below the non-inferiority limit, non-inferiority will be concluded. For the primary non-inferiority hypothesis, we will perform a per protocol analysis and an intention to treat analysis as sensitivity analysis.

#### Secondary study parameter(s)

The proportion of patients with a surgical site infection will be compared between groups using Pearson’s chi-squared test. Differences in the mean scores on the SF-12 and the NRS for pain and for cosmesis will be compared using the independent-samples t-test. Poisson regression will be used to compare the number of outpatient department visits, measured during the first 6 months post-operatively. All secondary parameters will be tested on the intention to treat sample, and are measured over the first 6 months after treatment.

### Monitoring

No formal data monitoring committee was formed as the expected risk for participants and for data collection and processing is minimal. However, the trial will be periodically monitored by an external, independent monitoring organ (CTCM, Clinical Trial Center Maastricht) to evaluate trial conduct, such as quality and completeness of the data, patient follow-up, and identify if timely adjustments need to be made. Adverse events are defined as any undesirable experience occurring to a subject during the study, whether or not considered related to the trial procedure. All adverse events from grade 3 or higher according to the Common Terminology Criteria for Adverse Events grading (CTCAE-grading, Version 4.0, 2009) which occur within 6 months postoperatively reported by the subject or observed by the investigator or his staff will be recorded. An adverse event that meets the criteria for a serious adverse event (SAE) will be reported by the investigator to the accredited medical-ethical committee that approved the protocol according to guidelines.

### Ethics and dissemination

The study will be conducted according to the principles of the Declaration of Helsinki (Version 2008, December 2018) and in accordance with the Medical Research Involving Human Subjects Act (WMO) and other guidelines, regulations and Acts. All eligible patients will be informed about the study in the outpatient clinic as soon as the indication for mastectomy has been made. They will be informed by their surgeon and will receive a document with information regarding the study as well as an informed consent form. Written informed consent will be obtained before patients are scheduled for surgery. Subjects can leave the study at any time for any reason if they wish to do so without any consequences. Any amendments to the study protocol, which may impact the conduct of the study, will require a formal amendment to the local research ethics committee. Publications will follow international guidelines: CONSORT Statement. The results of the scientific research will be submitted to peer-reviewed journals and disclosed unreservedly.

## Discussion

Mastectomy with flap fixation is becoming standard practice and is currently combined with closed-suction drainage. The current standardized use of closed suction drainage is supported by trials showing a higher frequency of seroma and greater volumes of seroma in patients undergoing mastectomy without drains [19–23]. However, in these studies no flap fixation was performed. Retrospective trials conducted comparing flap fixation with sutures with and without drainage found promising results in omitting drainage following mastectomy and concluded that it is safe to avoid drainage postmastectomy [15, 32]. These results were supported by the results of a randomized controlled trial performed by Purushotham et al. (2002) [33]. However, this trial compared conventional wound closure with a drain in the pectoral area and flap fixation without a drain in the pectoral area. All patients had a drain in the axillary area; thus, drainage was not completely omitted. Jain et al. (2004) performed a randomized controlled trial where drainage was omitted completely [24]. Though, in this study conventional wound closure with drainage was compared without drainage and the group without drainage was split in conventional wound closure and flap fixation with tissue glue.

To our knowledge, no randomized controlled trial has been conducted comparing flap fixation with and without closed-suction drainage with seroma aspiration as the primary outcome. The results of this trial could lead to omitting closed-suction drainage in mastectomy.

## Data Availability

The datasets used and/or analyzed during this study are not publicly available due to protection of the rights and privacy of the patients, but will be available from the corresponding author on reasonable request.

## Abbreviations

CCMO: Central Committee on research involving human subjects
CONSORT: CONsolidated standards of reporting trials
CRF: Case report form
CTCAE: Common terminology criteria for adverse events
DCIS: Ductal carcinoma in situ
MCS: Mental component summary
METC: Medical ethics committee
NRS: Numeric rating scale
PCS: Physical component summary
SAE: Serious adverse event
SARA: Seroma reduction and drain free mastectomy
SF-12: Short form 12
SSI: Surgical site infection
WMO: Wet medisch-wetenschappelijk onderzoek met mensen (medical research involving human subjects act)

## References

[1] Kumar S, Lal B, Misra MC (1995). Post-mastectomy seroma: a new look into the aetiology of an old problem. J R Coll Surg Edinb.

[2] Woodworth PA, McBoyle MF, Helmer SD, Beamer RL (2000). Seroma formation after breast cancer surgery; incidence and predicting factors. Am Surg.

[3] Carless PA, Henry DA (2006). Systematic review and meta-analysis of the use of fibrin sealant to prevent seroma formation after breast cancer surgery. Br J Surg.

[4] Agrawal A, Ayantunde AA, Cheung KL (2006). Concepts of seroma formation and prevention in breast cancer surgery. ANZ J Surg.

[5] Van Bemmel AJ, van de Velde CJ, Schmitz RF, Liefers GJ (2011). Prevention of seroma formation after axillary dissection in breast cancer: a systematic review. Eur J Surg Oncol.

[6] Kuroi K, Shimozuma K, Taguchi T, Imai H, Yamashiro H, Ohsumi S, Saito S (2006). Effect of mechanical closure of dead space on seroma formation after breast surgery. Breast Cancer.

[7] Gong Y, Xu J, Shao J, Cheng H, Wu X, Zhao D, Xiong B (2010). Prevention of seroma formation after mastectomy and axillary dissection by lymph vessel ligation and dead space closure: a randomized trial. Am J Surg.

[8] Van Bastelaar J, Beckers A, Snoeijs M, Beets G, Vissers Y (2016). Flap fixation reduces seroma in patients undergoing mastectomy: a significant implication for clinical practice. World J Surg Oncol.

[9] Van Bastelaar J, van Roozendaal L, Granzier R, Beets G, Vissers Y (2018). A systematic review of flap fixation techniques in reducing seroma formation and its sequelae after mastectomy. Breast Cancer Res Treat.

[10] Van Bastelaar J, Theunissen LLB, Snoeijs M, Beets G, Vissers Y (2017). Flap fixation using tissue glue or sutures appears to reduce Seroma aspiration after mastectomy for breast Cancer. Clin Breast Cancer.

[11] Granzier RWY, van Bastelaar J, van Kuijk SMJ, Hintzen KFH, Heymans C, Theunissen LLB, van Haaren ERM, Janssen A, Beets GL, Vissers YLJ (2019). Reducing seroma formation and its sequelae after mastectomy by closure of the dead space: the interim analysis of a multi-center, double-blind randomized controlled trial (SAM trial). Breast..

[12] Ko E, Han W, Cho J, Lee JW, Kang SY, Jung SY, Kim EK, Hwang KT, Noh DY (2009). Fibrin glue reduces the duration of lymphatic drainage after lumpectomy and level II or III axillary lymph node dissection for breast cancer: a prospective randomized trial. J Korean Med Sci.

[13] Almond LM, Khodaverdi L, Kumar B, Coveney EC (2010). Flap anchoring following primary breast Cancer surgery facilitates early hospital discharge and reduces costs. Breast Care (Basel).

[14] Sakkary MA (2012). The value of mastectomy flap fixation in reducing fluid drainage and seroma formation in breast cancer patients. World J Surg Oncol.

[15] Ten Wolde B, van den Wildenberg FJ, Keemers-Gels ME, Polat F, Strobbe LJ (2014). Quilting prevents seroma formation following breast cancer surgery: closing the dead space by quilting prevents seroma following axillary lymph node dissection and mastectomy. Ann Surg Oncol.

[16] Benevento R, Santoriello A, Pellino G, Sciaudone G, Candilio G, De Fatico GS, Selvaggi F (2014). The effects of low-thrombin fibrin sealant on wound serous drainage, seroma formation and length of postoperative stay in patients undergoing axillary node dissection for breast cancer. A randomized controlled trial. Int J Surg.

[17] Eichler C, Fischer P, Sauerwald A, Dahdouh F, Warm M (2016). Flap adhesion and effect on postoperative complication rates using Tissuglu® in mastectomy patients. Breast Cancer..

[18] Vasileiadou K, Kosmidis C, Anthimidis G, Miliaras S, Kostopoulos I, Fahantidis E (2017). Cyanoacrylate adhesive reduces Seroma production after modified radical mastectomy or Quadrantectomy with lymph node dissection-a prospective randomized clinical trial. Clin Breast Cancer..

[19] Somers RG, Jablon LK, Kaplan MJ, Sandler GL, Rosenblatt NK (1992). The use of closed suction drainage after lumpectomy and axillary node dissection for breast cancer. Ann Surg.

[20] Soon PS, Clark J, Magarey CJ (2005). Seroma formation after axillary lymphadenectomy with and without use of drains. Breast.

[21] Baker E, Piper J (2017). Drainless mastectomy: is it safe and effective?. Surgeon..

[22] George S Stoyanov, Dragostina Tsocheva, Katerina Marinova, Emil Dobrev, Rumen Nenkov. Drainage after Modified Radical Mastectomy - A Methodological Mini-Review. Cureus. 2017;9(7):e1454. 10.7759/cureus.1454. 10.7759/cureus.1454 PMC5590707 28929038

[23] Zaidi S, Hinton C (2017). Breast cancer surgery without suction drainage and impact of mastectomy flap fixation in reducing seroma formation. Eur J Surg Oncol.

[24] Jain PK, Sowdi R, Anderson AD, MacFie J (2004). Randomized clinical trial investigating the use of drains and fibrin sealant following surgery for breast cancer. Br J Surg.

[25] Taylor JC, Rai S, Hoar F, Brown H, Vishwanath L (2013). Breast cancer surgery without suction drainage: the impact of adopting a ‘no drains’ policy on symptomatic seroma formation rates. Eur J Surg Oncol.

[26] Troost MS, Kempees CJ, de Roos MAJ (2015). Breast cancer surgery without drains: no influence on seroma formation. Int J Surg.

[27] Barwell J, Campbell L, Watkins RM, Teasdale C (1997). How long should suction drains stay in after breast surgery with axillary dissection?. Ann R Coll Surg Engl.

[28] Gupta R, Pate K, Varshney S, Goddard J, Royle GT (2001). A comparison of 5-day and 8-day surgical drainage following mastectomy and axillary clearance. Eur J Surg Oncol.

[29] Kottayasamy Seenivasagam R, Gupta V, Singh G (2013). Prevention of seroma formation after axillary dissection--a comparative randomized clinical trial of three methods. Breast J.

[30] Chan AW (2013). SPIRIT 2013 statement: defining standard protocol items for clinical trials. Ann Intern Med.

[31] Chan AW (2013). SPIRIT 2013 explanation and elaboration: guidance for protocols of clinical trials. BMJ.

[32] Ouldamer L, Caille A, Giraudeau B, Body G (2015). Quilting suture of mastectomy dead space compared with conventional closure with drain. Ann Surg Oncol.

[33] Purushotham AD, McLatchie E, Young D, George WD, Stallard S, Doughty J, Brown DC, Farish C, Walker A, Millar K, Murray G (2002). Randomized clinical trial of no wound drains and early discharge in the treatment of women with breast cancer. Br J Surg.

